# All that is silver is not toxic: silver ion and particle kinetics reveals the role of silver ion aging and dosimetry on the toxicity of silver nanoparticles

**DOI:** 10.1186/s12989-018-0283-z

**Published:** 2018-12-05

**Authors:** Jordan N. Smith, Dennis G. Thomas, Hadley Jolley, Vamsi K. Kodali, Matthew H. Littke, Prabhakaran Munusamy, Donald R. Baer, Matthew J. Gaffrey, Brian D. Thrall, Justin G. Teeguarden

**Affiliations:** 1 Health Effects and Exposure Science, Pacific Northwest National Laboratory, Richland, WA 99352 USA; 2 The Environmental and Molecular Sciences Laboratory, Pacific Northwest National Laboratory, Richland, WA 99352 USA; 3 Department of Environmental and Molecular Toxicology, Oregon State University, Corvallis, OR 93771 USA

**Keywords:** Nanoparticle, Dissolution, ISDD, ISD3, Dosimetry

## Abstract

**Background:**

When suspended in cell culture medium, nano-objects composed of soluble metals such as silver can dissolve resulting in ion formation, altered particle properties (e.g. mass, morphology, etc.), and modulated cellular dose. Cultured cells are exposed not just to nanoparticles but to a complex, dynamic mixture of altered nanoparticles, unbound ions, and ion-ligand complexes. Here, three different cell types (RAW 264.7 macrophages and bone marrow derived macrophages from wild-type C57BL/6 J mice and Scavenger Receptor A deficient (SR-A^(−/−)^) mice) were exposed to 20 and 110 nm silver nanoparticles, and RAW 264.7 cells were exposed to freshly mixed silver ions, aged silver ions (ions incubated in cell culture medium), and ions formed from nanoparticle dissolution. The In Vitro Sedimentation, Diffusion, Dissolution, and Dosimetry Model (ISD3) was used to predict dose metrics for each exposure scenario.

**Results:**

Silver nanoparticles, freshly mixed ions, and ions from nanoparticle dissolution were toxic, while aged ions were not toxic. Macrophages from SR-A^(−/−)^ mice did not take up 20 nm silver nanoparticles as well as wild-types but demonstrated no differences in silver levels after exposure to 110 nm nanoparticles. Dose response modeling with ISD3 predicted dose metrics suggest that amount of ions in cells and area under the curve (AUC) of ion amount in cells are the most predictive of cell viability after nanoparticle and combined nanoparticle/dissolution-formed-ions exposures, respectively.

**Conclusions:**

Results of this study suggest that the unbound silver cation is the ultimate toxicant, and ions formed extracellularly drive toxicity after exposure to nanoparticles. Applying computational modeling (ISD3) to better understand dose metrics for soluble nanoparticles allows for better interpretation of in vitro hazard assessments.

## Background

Continued growth in the number and use of nano-objects including nanoparticles in consumer products has maintained demand for conventional and high-throughput in vitro approaches for hazard identification. Particles have unique properties that influence particle transport, dissolution, and intracellular fate, which are important considerations for in vitro cellular dose [1–8]. In addition, high levels of proteins and salts commonly found in cell culture media can induce changes in physicochemical properties of nanoparticles [9]. These changes influence the nature and extent of interactions between particles and cells. Dissolved salts can alter particle coatings and surface charge inducing agglomeration changing the effective size and density of particles [2, 7]. Nano-object size, shape, effective density, medium density, viscosity, and temperature will affect how nanomaterials move through medium by diffusion and sedimentation processes [6]. As a result, variations in these particle or system characteristics can lead to differences in the fraction of administered particle doses reaching cells residing at the bottom of a cell culture plate. Increasing the height of the cell culture medium, for example, can change the number, mass, and/or surface area of particles reaching cells by increasing the distance a particle has to travel to reach cells in given time [4]. Proteins and other medium constituents bound to particle surfaces create a corona that can modulate both cellular uptake through cell-surface receptors and resulting biological response [10, 11].

Soluble nanoparticles, commonly formed from metals such as copper, zinc, silver, manganese, and cerium, release ions in solution as they dissolve, reducing the mass of the particle. The extent of particle dissolution can depend on the material and its form. For example, 20 nm silver nanoparticles synthesized on gold seed particles have smaller crystallite size, more high-energy grain boundaries and defects, and higher apparent solubility compared to pure silver nanoparticles cohorts [12]. Ions can bind with ligands (e.g. other counter ions and proteins) present in cell culture medium, creating a mixture of metal ion-ligand complexes [13–15]. Some of these ion-ligand complexes may be formed in amounts that would exceed saturation limits, leading to nucleation and formation of precipitates and/or new nanoparticles [12, 14, 15]. For soluble or partially soluble particles, dissolution, sedimentation and diffusion within a cell culture system can lead to cells being exposed to a dynamic, complex mixture of nanoparticles, ions, and ion-ligand complexes.

Silver nanoparticles in aqueous systems are an example of this complex mixed particle and ion exposure system. Silver is found in 30% of consumer products registered in nano-product databases, and colloidal silver biocides have been registered for use in the United States since the 1950s [16, 17]. An estimated 20 tons of silver nanomaterials was produced in the United States during 2010 [18]. Exposure to silver nanoparticles can induce inflammatory responses, oxidative stress, and cytotoxicity in cultured cells [19]. Under standard cell culture conditions, silver nanoparticles can agglomerate and dissolve [12–15, 20], exposing cells to a complex mixture of nanoparticles, ions, and ion-ligand complexes with cell culture medium constituents. In cell culture medium, 20 nm silver nanoparticles > 9 μg/mL can result dissolved silver concentrations > 1 μg/mL within 1 h, indicating that silver ion exposures to cultured cells is significant following nanoparticle exposures [12]. Due to the complex nature of silver nanoparticle exposure, there remains uncertainty, and to some extent, controversy, regarding the extent to which each constituent—ion, ion-protein complex, particle—contributes to cellular toxicity [17, 19, 21]. For example, some have hypothesized a “Trojan-Horse” mechanism, where internalized nanoparticles undergo rapid dissolution resulting in silver ions inducing toxicity [22–26]. Others suggest that exposure to silver ions formed extracellularly are responsible for observed toxic effects [21, 27, 28]. Deconvoluting roles of particles and ions to test these hypotheses requires experimental designs and, in some cases, supportive modeling methods that yield cellular measures of exposure to both constituents in realistic test conditions.

If in vitro systems are to be relied upon to accurately rank nanomaterial hazards, study mechanisms of action, or conduct in vitro to in vivo extrapolation of dosimetry, understanding and measuring cellular dosimetry is important to understanding and measuring response [2, 6]. The objective of this work was to elucidate the separate roles of silver ions and particles in the induction of cellular toxicity by integrating toxicity testing, with experimental dosimetry and biokinetic modeling. Cellular viability studies were conducted with three types of macrophages using two silver particles with different dissolution rates, 20 and 110 nm, and silver ions (indirectly formed from particles and silver acetate). Scavenger receptor A (SR-A) competent (wild-type) and SR-A deficient (−/−) macrophages were used to control for the role of particle uptake on cellular content and toxicity [25, 29]. Particle and ion cellular dosimetry was calculated using the In Vitro Sedimentation, Diffusion, Dissolution, and Dosimetry Model (ISD3) (See companion paper, [20]). ISD3 calculates the time course of silver particle dissolution and cellular concentrations of ions and particles under the cell culture conditions used here. Previously, our group demonstrated that silver nanoparticle dissolution depends on time, nanoparticle composition, nanoparticle surface area, cell culture medium, and amount of protein in cell culture medium [12, 20]. Measured silver nanoparticle dissolution rates along with uptake data in cells were used to parameterize ISD3 [20]. Dose response modeling was used to determine the comparative potency of silver ions and particles and correlate cellular doses with toxicity. The coordinated application of dosimetry and biokinetic modeling with toxicity testing revealed differential potency of aged and unaged silver ions and a consistent dose-response for silver toxicity across concentration, particle size in macrophage cells with normal capacity for particle uptake, and cells deficient in SR-A with a reduced capacity for particle uptake. This work demonstrates the importance of understanding and measuring dosimetry for effective use of in vitro test systems for toxicity testing.

## Methods

### Chemicals

RPMI 1640 Medium was obtained from Gibco Life Technologies (Grand Island, NY, USA). Fetal bovine serum (FBS) was purchased from Atlanta Biologicals (Flowery Branch, GA, USA). L-glutamine and Pen-Strep were purchased from Invitrogen (Grand Island, NY, USA). Double distilled concentrated hydrochloric and nitric acids were obtained from GFS Chemicals, Inc. (Columbus, OH, USA). Silver acetate (99.99%) and other general laboratory chemicals were acquired from Sigma-Aldrich (St. Louis, MO, USA).

### Nanoparticles

Citrate-coated silver particles with primary diameters of 20 (lot number MGM 1659) and 110 nm (lot numbers MGM 1662) containing a gold core of 7 nm manufactured by nanoComposix (San Diego, CA, USA) at a concentration of 1 mg/mL (PNNL arrival date 11/28/11) were provided by the National Institute of Environmental Health Sciences (NIEHS) Centers for Nanotechnology Health Implications Research (NCNHIR). These particles were reported to have hydrodynamic diameters of 24 and 104 nm, respectively, in water by the Nanotechnology Characterization Laboratory (NCL) using Dynamic Light Scattering (DLS) with a Malvern Zetasizer Nano ZS instrument (Southborough, MA, USA) and core diameters of 20.3 and 111.5 nm by Transmission Electron Microscopy (TEM). Nanoparticle stocks were stored in the dark at 4 C until utilized.

### Nanoparticle characterization

Hydrodynamic diameters of silver nanoparticles in RPMI were measured using DLS with a ZetaPALS zeta potential and particle size analyzer (Brookhaven Instruments Corporation, Holtsville, NY, USA). Hydrodynamic diameter of nanoparticles (100 μg/mL) was calculated from intensity weighted average translational diffusion coefficient using cumulant analysis on the autocorrelation function using vendor provided software. Stock suspensions of nanoparticles were tested for endotoxin levels using a Toxinsensor Chromogenic LAL kit (GenScript, Piscataway, NJ, USA). Substantial characterization of these nanoparticles has been previously reported including quantified nanoparticle dissolution and agglomeration, structural feature analysis of nanoparticle dissolution using both scanning/transmission electron microscopy (S/TEM) and high resolution TEM (HR-TEM) imaging, and spectral analysis using X-ray photoelectron spectroscopy (XPS), [12, 20, 30, 31].

### Silver ion test solutions

Fresh and aged silver ion solutions were prepared independently for toxicity testing. Fresh solutions were generated by mixing silver acetate with RPMI and 10% FBS and immediately exposing cells. “Aged” silver ion solutions were prepared by mixing with RPMI and 10% FBS and incubating for 0, 0.5, 1, 3, 6, or 24 h before use. To generate solutions of ions formed from particles, silver nanoparticles (50 μg/mL, 20 nm, coated in citrate), were incubated in cell culture medium for 6 h at standard cell culture conditions (*n* = 3). Since Munusamy et al. [12] observed rates of silver nanoparticle dissolution are dependent on nanoparticle concentration and surface area, 20 nm nanoparticles were chosen to provide maximum dissolved silver ions in a 6 h period. Nanoparticle suspensions were then centrifuged at 30,000 rpm (49,000×g maximum, 38,000×g average, and 27,000×g minimum) for 90 min as with previous dissolution studies [12]. Supernatants were collected, serially diluted, and dosed to RAW 264.7 cells. Silver levels in supernatants and dilutions were quantified using inductively coupled plasma-mass spectrometry (ICP-MS). Cells were assayed for toxicity (see *Cellular Viability*).

### Animals

Wild-type C57BL/6 J mice were acquired from Jackson Laboratory (Sacramento, CA, USA) and were housed individually in standard rodent cages. SR-A knockout [SR-A^(−/−)^] C57BL/6 J mice were acquired from the University of Washington (Seattle, WA, USA) breeding colony. Breeding pairs of SR-A^(−/−)^ were originally acquired from Jackson Laboratory and bred at the University of Washington Transgenic Animal Facility. Water and feed (PMI 5002, Certified Rodent Diet) were provided ad libitum. All procedures involving animals were in accordance with protocols established in the NIH/NRC Guide and Use of Laboratory Animals (NIH/NRC) and were reviewed by the Institutional Animal Care and Use Committee of Battelle, Pacific Northwest Division.

### Isolation and culture of cells

Macrophages play an important role in nanoparticle clearance and potential toxic effects of nanoparticles [32]. Macrophages were used to parameterize cellular uptake parameter within ISD3 [20]. As such, primary and immortalized macrophages were used as cellular models in this study.

Wild-type and SR-A^(−/−)^ mice were euthanized using CO_2_ asphyxiation, and femurs were removed and cleaned of muscle and connective tissue. Primary bone marrow cells were flushed from femurs using 5 mL of RPMI 1640 with 2 mM L-glutamine, 100 U/mL Pen-Strep, and 10% fetal bovine serum using a 25-gauge needle into a 50 mL centrifuge tube on ice. Isolated cells were centrifuged and cultured in 100 cm^2^ dishes with 10 mL RPMI 1640 supplemented with L-glutamine, Pen-Strep, 10% FBS, and 20% L929 conditioned medium. Every 2 days, cells were washed with PBS to remove non-adherent cells, and medium was replaced. Seven days after isolation, bone marrow derived macrophages were ready for use in the experiment. Conditioned medium was made by growing L929 cells to confluence in RPMI 1640 supplemented with L-glutamine, Pen-Strep, and 10% FBS for 7 days. The supernatant was collected, centrifuged to remove cellular debris, and filtered (0.2 μm). Fresh medium was added, and cells were grown for an additional week. The supernatant was collected and processed as previously described. Media from both weeks was pooled for use in bone marrow cell differentiation. Stocks of conditioned medium were aliquoted and stored at − 20 C until use.

Mouse alveolar macrophage cells (RAW 264.7, ATCC # TIB 71) were grown at standard cell culture conditions (37 C, ~ 5% CO_2_) and seeded in 6-well or 96-well plates in RPMI 1640 supplemented with L-glutamine, Pen-Strep, and 10% FBS.

### Cellular viability

Viability of three cell types, RAW 264.7 cells and bone marrow macrophages from SR-A^(−/−)^ and wild-type mice were evaluated after exposure to nanoparticles. Cells were exposed to 20 or 110 nm silver nanoparticles at suspension concentrations of 6.25, 12.5, 25 or 50 μg/mL (0.1 mL) in 96-well plates (*n* = 3). In vitro dose solutions were prepared immediately prior to dosing by mixing stock nanoparticle suspension with FBS and diluting with the cell culture medium. Cells were incubated with nanoparticles for 24 h at standard cell culture conditions, and cell viability was assessed.

RAW 264.7 cells were dosed with 0.9–2.8 μg/mL silver ions (from silver acetate) mixed freshly in cell culture medium. Cells were incubated for 24 h at standard cell culture conditions, and cell viability was assessed.

RAW 264.7 cells were dosed with solutions (2.5 and 5.0 μg/mL) of silver ions aged for 0.5, 1, 3, 6 or 24 h, or solutions of ions from dissolved particles for 24 h at standard cell culture conditions. At that time, viability was assessed.

All ion toxicity experiments were conducted in the same sized plates and volumes as the nanoparticle toxicity assays.

Membrane damage was used as a surrogate for cellular viability measured using the lactate dehydrogenase (LDH) assay. LDH was measured using CytoTox-ONETM membrane integrity assay (Promega, Madison, WI, USA). Briefly, an aliquot (100 μL) of supernatant from lysed and non-lysed replicates (*n* = 3 each) were assayed using reagents provided in the kit. Fluorescence (excitation at 560 nm and emission at 590 nm) was measured using a spectrofluorometer (Cytofluor 4000, Perseptive Applied Biosystems, Cambridge, MA, USA). Viability was calculated as difference between the lysed and non-lysed replicates of treated divided by the same calculation in non-treated controls. Confidence intervals were calculated by bootstrapping viability calculations using random sampling with replacement.

### In vitro cellular uptake

Uptake of silver nanoparticles was assessed in three cell types. Cells were cultured in 6-well plates at standard cell culture conditions (37 C, ~ 5% CO^2^) and exposed to 12.5 μg/mL of 20 or 110 nm silver nanoparticle suspensions (3 mL). After exposure, cells were incubated for 0.5–24 h. After incubation, cells were washed and scraped. A small aliquot (10 μL) was collected for cell counting using a hematocytometer. Total silver levels in remaining cells were quantified using ICP-MS.

### Silver quantification

Silver levels in cell culture medium and cells were quantified using ICP-MS. Samples were spiked with ^89^Y as an internal standard and digested with 70% double distilled nitric acid (~ 2 mL) overnight until clear. Afterwards, double distilled concentrated hydrochloric acid (~ 1 mL) was added to shift the equilibrium from insoluble silver to soluble silver chloride complexes. Aliquots were diluted to 2% nitric acid, and total silver was quantified using an Agilent 7500 CE (Santa Clara, CA, USA) inductively coupled plasma-mass spectrometer. ^107^Ag measured in helium collision mode using ^45^Sc and ^115^In (10 ng/mL) as internal standards. Additionally, ^109^Ag was also monitored. Three rinses with 2% nitric acid between runs were used to minimize silver carryover. Quantification was accomplished using a linear regression fit to an external calibration curve. The calibration curve was made by spiking silver standards (VHG Labs, Inc., Manchester, NH, USA) in either cell culture medium or cells, depending on the sample matrix, and processed simultaneously with the samples. Limits of quantification for silver were ~ 0.1 ng/mL for samples diluted to 2% nitric acid.

### Computational silver ion and particle Dosimetry

ISD3 was used to calculate cellular doses of silver particles and silver ions for each exposure scenario [20]. The model was calibrated to measured ion and total silver uptake time-course data for multiple concentrations of the 20 and 110 nm silver nanoparticles [20]. For simulations of 20 nm nanoparticle exposures to SR-A^(−/−)^ cells, cellular uptake was reduced to accurately describe silver levels in cells for that exposure scenario (see **Results**). ISD3 was then used to simulate all nanoparticle toxicity exposures. ISD3 was coded and implemented in Matlab.

### Dose response modeling

Loss of cell viability as a function of various dose metrics calculated using ISD3 for each exposure scenario were evaluated using dose response modeling. A Hill Equation (Eq. 1, [33]) was used to describe loss of cell viability (*LV*) as a function of ISD3 predicted dose metrics (*x*) after various exposure scenarios, where *h* is the Hill Coefficient and *b* is the median lethal dose metric (LD_50_).

1 $$ LV(x)=\frac{1\times {x}^h}{x^h+{b}^h} $$

Parameter optimizations were achieved using a maximum log likelihood objective with the Quasi-Newton Method algorithm. Akaike information criterion (AIC) was used for model selection. Software used to analyze data was “R: A language and environment for statistical computing”, version 3.2.3 (Vienna, Austria).

## Results

### Nanoparticle characterization

Stock suspensions of nanoparticles tested negative for endotoxin (< 0.01 EU/mL). The effective hydrodynamic diameter of nanoparticles increased from the primary size after being prepared for administration to cells, and zeta potentials were negative (Table 1). These are similar to agglomerate sizes and zeta potentials measured in previous studies using similar conditions [12].

**Table 1:** Primary diameter, effective diameter, and zeta potential of silver nanoparticles used in this study

Primary Diameter (nm)	Effective Diameter (nm)	Zeta Potential (mV)
20	44	−7.7
110	155	−9.8

### Cellular silver Dosimetry for dose-response analysis

Two approaches were used to deconvolute roles of silver particles and silver ions in silver nanoparticle toxicity during mixed exposures typical of in vitro test systems. First, the contribution of particles to toxicity and cellular doses was evaluated experimentally by modulating particle uptake using SR-A-competent and SR-A-deficient (SR-A^(−/−)^) cells. Second, cellular doses of silver particles and silver ions were calculated using ISD3, a computational model of silver particle dissolution, particle delivery, and ion uptake calibrated using data from the experimental system used for studies in this manuscript (see Companion Paper, [20]).

### Effects of modulating particle update on silver cellular Dosimetry

**Fig. 1:**
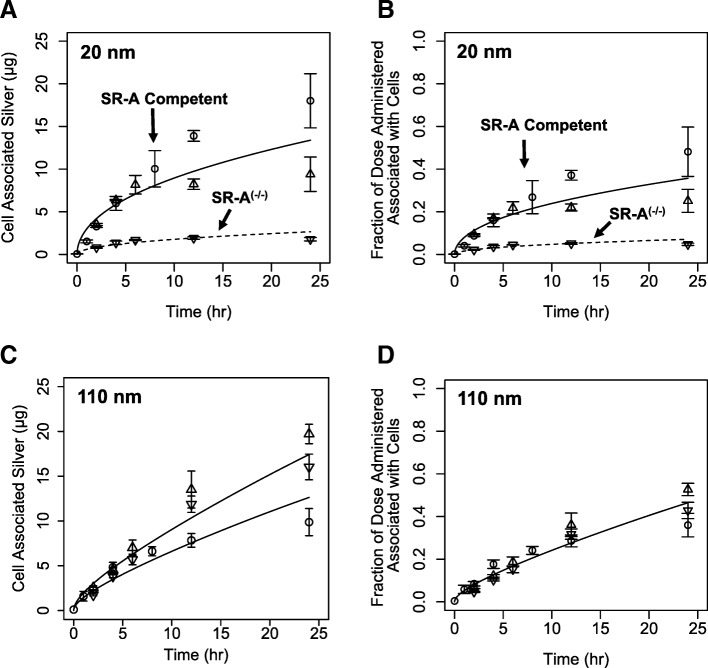
Time course of cell associated silver amounts in RAW 264.7 cells (circle) and bone marrow derived macrophages from wild-type mice (up triangle) and SR-A deficient mice (down triangle) exposed to a nominal concentration of 12.5 μg/mL of 20 nm (**a-b**) or 110 nm (**c-d**) silver nanoparticles. RAW 264.7 cells were exposed to measured exposure concentration of 9.15 μg/ml 110 nm silver nanoparticles (**c**). Solid lines are unmodified ISD3 simulations of silver amounts in cells from measured exposures. The dotted line is the cellular silver content calculated using ISD3 adjusted for reduced uptake of particles reaching SR-A deficient cells exposed to 20 nm silver nanoparticles

Cells with differing levels of SR-A proficiency demonstrated different patterns of silver uptake after exposure to 20 nm nanoparticles but similar silver levels after exposure to 110 nm nanoparticles after exposure to 12.5 μg/mL nominal nanoparticle concentrations. Total silver amounts in SR-A competent bone marrow macrophages were not significantly different (*p* > 0.06) from corresponding silver amounts in SR-A competent RAW 264.7 cells until 12 h post exposure (*p* < 0.02) to 20 nm silver nanoparticles (Fig. 1a-b). Lower total silver amounts in bone marrow macrophages at later time points may reflect reduced uptake due to differences in sensitivity to silver, or cell-specific saturation points for uptake. Total silver associated with cells following exposure to 20 nm silver nanoparticles were 6–10 fold lower in bone marrow macrophages from SR-A^(−/−)^ mice compared to the corresponding SR-A^(+/+)^ bone marrow macrophages and SR-A competent RAW 264.7 cells (Fig. 1a-b). This data is consistent with the hypothesis that total cellular silver is composed primarily of silver nanoparticles, and uptake of 20 nm particles is in-part SR-A dependent. In contrast, exposure to 110 nm particles resulted in total cellular silver levels (normalized to administered dose) in SR-A-competent and SR-A-deficient cell types that were not significantly different until 24 h (Fig. 1c-d). This suggests that SR-A may not play a prominent role in 110 nm nanoparticle uptake as observed with 20 nm silver nanoparticles. The differential silver content of SR-A competent and deficient macrophages under similar mixed particle and ion exposures provides a unique data set for evaluating separate roles of silver nanoparticles and silver ions on cell viability.

### Total cellular silver, silver ion and silver nanoparticle doses

The ISD3 particokinetic model for soluble silver nanoparticles [20] was used to calculate the time course of cellular silver ion and particle doses. ISD3 was calibrated to silver nanoparticle dissolution and silver ion cellular partitioning data in RAW 264.7 cells [20]. Total silver cellular dose is the sum of silver ions partitioned into cells and particles delivered by sedimentation or diffusion to the cell surface. Assuming that all silver nanoparticles taken up by cells upon arrival, ISD3 accurately calculated the full time course of total cellular silver for all three cell types exposed to 110 nm silver particles (Fig. 1c) and 20 nm particle uptake competent RAW 264.7 cells and wild-type bone marrow macrophages (Fig. 1a). In contrast with the same uptake assumption, ISD3 calculated total silver content of the SR-A^(−/−)^ cells exposed to 20 nm nanoparticles significantly higher than measured values (Fig. 1a). This is consistent with reduced particle uptake expected in SR-A^(−/−)^ cells. Adjusting the assumed particle uptake fraction from 100 to 20% resulted in improved predictions of total cellular silver levels. ISD3 calculated cellular doses for all cell types exposed to 20 and 110 nm particles indicated that < 1–2% of total cellular silver is in the form of ions. Due to this observed differential nanoparticle uptake, these cell systems were used as models for evaluating separate roles of silver ions and particles in toxicity.

### Mixed silver ion and particle toxicity

**Fig. 2:**
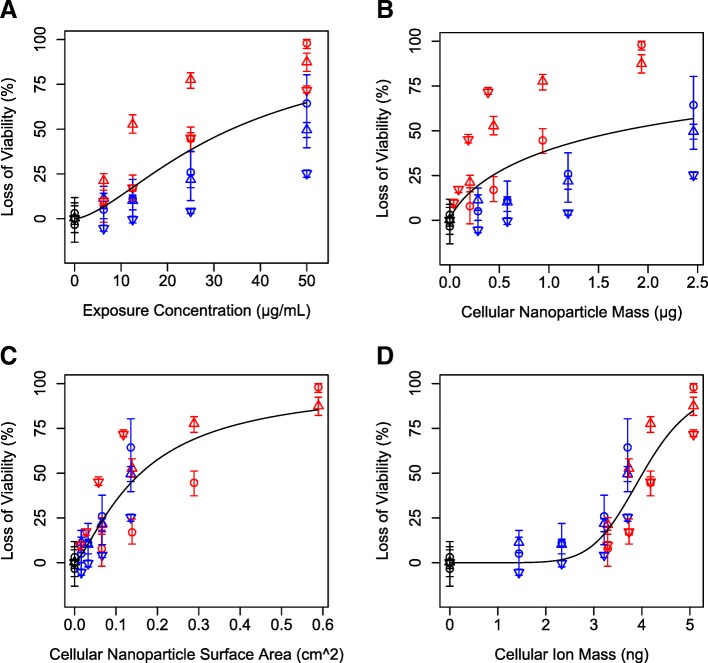
Loss of viability in RAW 264.7 cells (circle) and bone marrow derived macrophages from wild-type mice (up triangle) and SR-A deficient mice (down triangle) exposed to various concentrations of 20 nm (red) or 110 nm (blue) silver nanoparticles (**a**) as a function of various dose metrics predicted by ISD3 including cellular nanoparticle mass (**b**), cellular nanoparticle surface area (**c**), and cellular ion mass (**d**). Lines are dose response model fits to the data

Exposure to 20 or 110 nm silver nanoparticles and their dissolution products caused loss of viability in RAW 264.7 cells and bone marrow derived macrophages, with significantly different dose-response curves (Fig. 2), particularly for SR-A^(−/−)^ cells. We hypothesized that the form of silver causing toxicity could be identified by consistent LD_20_ values across particles and cell types. For example, if silver ions from cell culture medium entering cells were primary drivers of toxicity, similar LD_20_ values would be observed between particle uptake competent cells and SR-A^(−/−)^ cells. LD_20_ values for all cell types and particles based on ISD3 calculated silver ion content ranged from 3.0–3.8 ng, demonstrating the most consistent dose metric measured by coefficient of variation (10%; Table 2). LD_20_ values based on other dose metrics including total silver nominal media concentration, cellular nanoparticle mass, and cellular nanoparticle surface area were much more variable (coefficient of variation: 67–86%; Table 2). These findings support our hypothesis that silver ions derived from media were driving toxicity of silver nanoparticles, even under conditions of combined particle/ion exposures.

**Table 2:** Dose metrics causing 20% loss of viability (LD_20_) to (bone marrow derived macrophages from wild-type C57BL/6 J mice (WT) or Scavenger Receptor A deficient mice (SR-A (−/−) or RAW 264.7 (RAW) cells after exposure to silver nanoparticles of various sizes

		LD_20_			
Nanoparticle Size (nm)	Cell Type	Exposure Concentration (μg/mL)	Cellular Nanoparticle Mass (μg)	Cellular Nanoparticle Surface Area (cm^2^)	Cellular Ion Mass (ng)
20	RAW	15.9	0.93	0.18	3.82
20	WT	6.25	0.18	0.06	3.21
20	SR-A^(−/−)^	12.7	0.09	0.03	3.67
110	RAW	19.9	0.94	0.05	3.08
110	WT	20.2	0.95	0.05	3.02
110	SR-A^(−/−)^	45.3	2.22	0.12	3.62
CV^a^ (%)		67	86	70	10

^a^Coefficient of variation (CV)

### Fresh and aged silver ion toxicity

**Fig. 3:**
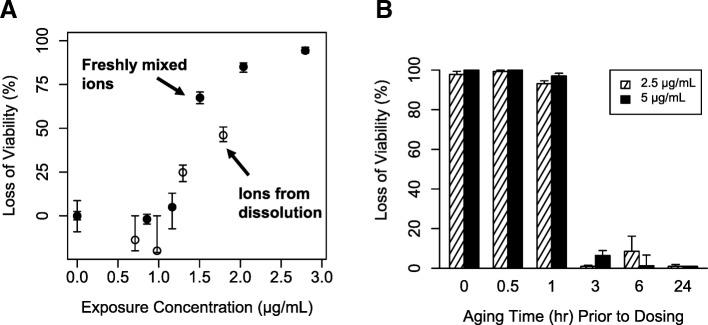
Loss of viability in RAW 264.7 cells exposed to freshly mixed silver ions (closed circle) and silver ions formed from dissolution of 20 nm silver nanoparticles (open circle) (**a**). Loss of viability in RAW 264.7 cells exposed to silver ions (2.5 or 5 μg/mL) for 24 h aged in cell culture medium for various amounts of time before dosing to cells (**b**). Loss of viability was assessed 24 h post exposure for all experiments

Unaged silver ions were 5–35 times more toxic on a nominal media concentration basis compared to silver nanoparticles (Figs. 2a, 3a). Freshly mixed silver ions produced a steep dose-response curve for cell viability in RAW 264.7 cells after 24 h of exposure (Fig. 3a). LD_20_ and LD_50_ values were very close (1.28 and 1.42 μg/mL), indicating a steep dose-response curve. Almost complete (98%) loss of viability was observed after 24 h of exposure to 2.4 μg/mL of freshly mixed silver ions. LD_20_ values from nanoparticle exposures ranged 6–45 μg/mL (Table 2).

Aging silver ions (incubating in cell culture medium) for three or more hours before dosing significantly attenuated toxicity in RAW 264.7 cells. Silver ions aged for ≤1 h prior to dosing caused almost complete loss of cellular viability after 24 h of exposure at two exposure concentrations (2.5 or 5.0 μg/mL; Fig. 3b). Nearly no toxicity was observed after aging ions for ≥3 h (Fig. 3b) under the same exposure conditions. We hypothesized that during aging, silver cations (Ag^+^) bind to ligands available in cell culture medium (e.g. Cl^−^, SO_4_^−^, S^−^, proteins, etc.), and these bound silver complexes are less toxic than unbound silver cations. We hypothesize that the abrupt change in toxic to nontoxic is due steep dose response curve for silver ions (Fig. 3a).

Silver ions formed from nanoparticle dissolution were toxic to RAW 264.7 cells (LD_50_: 1.81 μg/mL, Fig. 3a). Two doses of ions formed from nanoparticle dissolution demonstrated toxicity. The highest dose of silver ions formed from dissolution (maximum ion concentration from ion harvesting conditions) were less toxic than freshly mixed ions (LD_50_: 1.42 μg/mL), while at lower doses, toxicity was similar (Fig. 3a).

### Cellular toxicity is best predicted by cellular silver ion mass

**Fig. 4:**
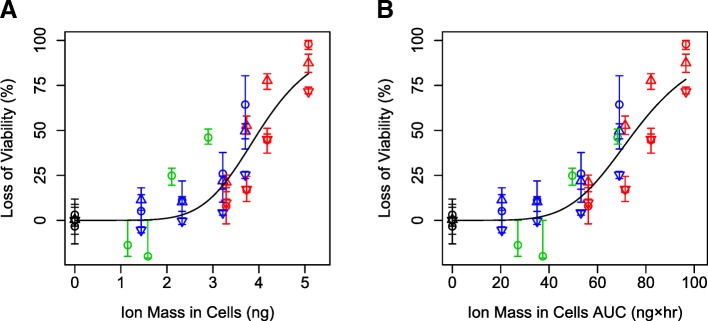
Loss of viability in RAW 264.7 cells (circle) and bone marrow derived macrophages from wild-type mice (up triangle) and SR-A deficient mice (down triangle) exposed to various concentrations of 20 nm (red), 110 nm (blue) silver nanoparticles, or silver ions formed from dissolution of 20 nm silver nanoparticles (green) as a function of ion mass in cells (**a**) or ion mass in cells AUC (**b**) predicted by ISD3. Lines are dose response model fits to the data

To further test our hypothesis that silver ions had the strongest influence of cellular toxicity, relationships between multiple dose metrics and viability were tested by fitting dose response models to data from all three cell types, both particles, and ion-only exposures combined into one dataset. ISD3 was used to calculate cellular dose metrics including intracellular particle mass, intracellular particle surface area, cellular ion mass, and associated AUCs. Intracellular nanoparticle mass poorly predicted loss of viability (highest AIC) (Table 3, Fig. 2b). This finding is consistent with a hypothesis that complete nanoparticle dissolution within cells is unlikely and not controlling toxicity. Intracellular ion mass and intracellular ion mass AUC were the best predictors of loss of cellular viability (lowest AIC’s) (Table 3). This finding is consistent with our hypothesis that silver ions are a primary contributor to cytotoxicity during exposure to silver nanoparticles in vitro, where ions and particles are both present. We found that the intracellular ion AUC was the best predictor of toxicity (AIC values: − 35.1 vs − 29.5, respectively; Fig. 4) when all dose-response data were included: ions only and mixed particle and ion exposures for 20 and 110 nm particles in three cell types.

**Table 3:** Akaike information criterion (AIC) values of dose response fits to cell viability as a function of various dose metrics of silver nanoparticle exposure

Dosimetric	Units	AIC
Nanoparticle Mass	μg	1.6
Nanoparticle Surface Area	cm^2^	−28.6
Ion Mass	μg	−35.0
Nanoparticle Mass AUC	μg × hr	−0.9
Nanoparticle Surface Area AUC	cm^2^ × hr	−28.5
Ion Mass AUC	μg × hr	−32.4
Exposure Concentration	μg/mL	−13.6

## Discussion

Cells are exposed to complex mixtures of ions, ion-ligands, and particles during silver nanoparticle exposures, making experimental determination of which form of silver that is driving observed cellular toxicity difficult. Two competing hypotheses propose that toxicity is a result of either extracellular [21, 27, 28] or intracellular (Trojan Horse) [22–26] nanoparticle dissolution. However, experiments directly testing these hypotheses are difficult, because measuring silver ions, silver ion-ligand species, and intracellular nanoparticle dissolution remain major challenges to the field of nanotoxicology. New technologies are emerging to address these challenges; however, most have limitations. X-ray absorption near-edge spectroscopy (XANES) has been suggested as a possible method for measuring various silver species [23, 34], but quantitative interpretation of XANES data is not well developed [35]. Cloud point extraction methods have been proposed as a method to measure silver ions and nanoparticles, but this method cannot yet differentiate between the silver cation and other silver ion-ligand species [36]. Fluorescent nanoparticle and ion labeling coupled with microscopy techniques have also been used [25], but these techniques require fluorescent tags to be bound to nanoparticles, resulting in modified nanoparticles used for experimental testing. Because of these experimental challenges, other approaches are needed to test hypotheses regarding the effects of intracellular and extracellular nanoparticle dissolution on cellular toxicity.

To overcome ion measurement challenges, we applied ISD3 as a tool to predict nanoparticle dose to cells, nanoparticle dissolution, and silver ion dose to cells for interpreting cytotoxicity in cells after exposure to silver nanoparticles and ions. ISD3 is an extension of our previously validated model for insoluble particles using first principles to describe diffusion and sedimentation of particles in in vitro systems [4]. ISD3 was developed for similar predictions with soluble particles, describing nanoparticle dissolution using a Population Balance Equation [20]. ISD3 was parameterized with nanoparticle dissolution and ion partitioning experiments using the same nanoparticles and cells used here [20]. Combining ISD3 simulations with routine, repeatable in vitro experiments allows a novel approach to differentiate roles of particles and ions on cellular toxicity.

SR-A^(−/−)^ cells were utilized as an additional experimental dimension to differentiate the effects of particles and ions on cellular toxicity. SR-A receptors display broad, overlapping ligand specificity, and previous studies have implicated SR-A with mediated nanoparticle uptake. After silencing levels of SR-A expression in RAW 264.7 and HEK-293 cells, decreased intracellular levels of anionic silica nanoparticles were observed compared to naïve RAW 264.7 cells using high resolution microscopy [29]. SR-A has demonstrated importance for silver nanoparticle uptake and trafficking [25]. Using gold nanoparticles, it was reported that SR-A mediated uptake for nanoparticles < 100 nm, while other mechanisms were responsible for larger nanoparticles [37]. Scavenger receptor B1 has also been demonstrated as a mechanism of uptake for 20 nm silver nanoparticles with bone marrow derived macrophages [38]. Overall, these published results are consistent with our findings that macrophages from SR-A^(−/−)^ mice did not take up 20 nm silver nanoparticles as proficiently as macrophages from wild-type mice, but both cell types demonstrated similar levels of uptake for 110 nm nanoparticles. Beyond mechanistic uptake implications of this observation, differential nanoparticle uptake allows for an experimental element to test the effects of extra- and intracellular nanoparticle dissolution.

Silver ion dose metrics were consistently the best predictors of cellular toxicity after silver nanoparticle and fresh silver ion exposures to all cell types. After exposing cells to silver nanoparticles or silver ions, we observed two different sizes of silver nanoparticles are toxic, freshly mixed silver ions are toxic, and aged silver ions are not toxic using three different cell types including a knockout of a known nanoparticle uptake mechanism. ISD3 was able to accurately predict silver levels in cells for each exposure scenario (Fig. 1). Across all exposure scenarios, except aged ions (which no toxicity was observed), silver ion mass AUC in cells best predicted toxicity in all cells regardless of their ability to take up nanoparticles (Fig. 4). Additionally, after all sizes of silver nanoparticle exposures to all cell types, a consistent level of ~ 3 ng silver ions in cells caused 20% loss of cellular viability (Table 2). Since ISD3 assumes that all silver ion levels in cells are from extracellular sources, these results support the hypothesis that silver ions formed extracellularly are responsible for observed toxicity.

Previous studies have reported cellular toxicity after exposure to silver ions formed by nanoparticle dissolution. Wang et al. [28] observed silver ions formed from nanoparticle dissolution were toxic to human bronchial epithelial (BEAS-2B) cells. Beer et al. [21] observed that silver nanoparticle suspensions were more toxic when the initial silver ion fraction was higher in A549 human lung carcinoma epithelial-like cells. Exposure to supernatant of centrifuged particles after incubation in cell culture medium for 24 h did not cause loss of cell viability, indicating ion aging had occurred [22]. After adding silver nitrate to cell culture medium, formation of silver chloride and silver sulfide precipitates were observed [26]. Our results are consistent with these previous studies and further demonstrate that aged silver ions (silver-ligand complexes) are much less toxic to cells than the unbound silver cation (Fig. 3). Also consistent with these previous studies, our results support the hypothesis that silver cations formed extracellularly from silver nanoparticle dissolution are toxic until bound with ligands.

Reports are conflicting of the extent of intracellular silver nanoparticle dissolution. For example, several studies have reported little to negligible levels of silver nanoparticles dissolution in artificial lysosomal fluid [22, 39], while others have reported marginal to much higher levels (~ 60–70% in 24 h) of intracellular dissolution [23, 25, 26, 40–42]. De Matteis et al. observed intracellular release of silver ions and diffusion of silver ions across the entire cell using a silver ion-specific florescent probe [42] and other systems [17]. Consistent with this observation, we modeled silver ion uptake into cells using Fick’s Diffusion, [20]. Silver nanoparticle dissolution rates are highly dependent on protein level (e.g. FBS) in both cell culture medium [12] and artificial lysosomal fluid [43]. Additionally, dissolution rates measured in our lab are dependent on nanoparticle concentration [12], which would be very high localized within lysosomes. These uncertainties make it challenging to quantify the extent of intracellular dissolution and accurately parametrize a computational model of dissolution with confidence. However, because of nanoparticle dissolution’s dependence on surface area [12, 20], we relied on intracellular surface area of nanoparticles as a metric for this process.

Intracellular dissolution of nanoparticles is a central tenant to the Trojan Horse hypothesis of nanoparticle toxicity [22–26]. While intracellular nanoparticle dissolution was not directly modeled here, we assume that this process is dependent on the intracellular surface area of nanoparticles in cells [12, 20]. Thus, we would expect intracellular nanoparticle surface area and associated AUC to be highly predictive of toxicity if intracellular dissolution was driving toxicity. We observed that intracellular ion metrics were better predictors of toxicity than intracellular nanoparticle surface area metrics, suggesting extracellular dissolution plays a more important role on cellular toxicity observed here compared to intracellular dissolution (Table 3).

## Conclusions

In conclusion, we observed silver nanoparticles and freshly mixed silver ions were toxic to cells, while aged silver ions were not toxic, implicating the unbound silver cation is the ultimate toxicant to cells after exposure to nanoparticles. ISD3 quantitatively described particle and ion dose metrics after nanoparticle exposure in cells with competent and deficient nanoparticle uptake. Dose response modeling with ISD3 simulated dose metrics suggest that amount of ions in cells and area under the curve (AUC) of ion amount in cells are the most predictive of cell viability after nanoparticle and combined nanoparticle/dissolution-formed-ions exposures, respectively. This observation implicates silver ions formed extracellularly drove toxicity after exposure to nanoparticles in this study. Applying computational modeling (ISD3) to better understand dose metrics for soluble nanoparticles allows for better interpretation of in vitro hazard assessments.
